# Clinical evaluation for the pharyngeal oxygen saturation measurements in shocked patients

**DOI:** 10.1186/s12912-022-01073-z

**Published:** 2022-11-01

**Authors:** Eman Arafa Hassan, Sherouk Nasser Mohamed, Emad Hamdy Hamouda, Nadia Taha Ahmed

**Affiliations:** 1 Critical Care and Emergency Nursing Department, Faculty of Nursing, Alexandria University, Alexandria, Egypt; 2 Critical Medicine Department, Faculty of Medicine, Alexandria University, Alexandria, Egypt

**Keywords:** Hypoxia, Oxygen saturation, Pharynx, Oximetry, Sensitivity, Shock, Specificity

## Abstract

**Background:**

Monitoring oxygen saturation in shocked patients is a challenging nursing procedure. Shock syndrome alters peripheral tissue perfusion and hinders peripheral capillary oxygen saturation (SpO2) measurements. Our study aimed to find a solution to this problem. The pharynx is expected to be an accurate SpO2 measurement site in shocked patients. We clinically evaluated the pharyngeal SpO2 measurements against the arterial oxygen saturation (SaO2) measurements.

**Methods:**

A prospective cohort research design was used. This study included 168 adult shocked patients. They were admitted to five intensive care units from March to December 2020 in an Egyptian hospital. A wrap oximeter sensor was attached to the posterior surface of an oropharyngeal airway (OPA) by adhesive tape. The optical component of the sensor adhered to the pharyngeal surface after the OPA insertion. Simultaneous pharyngeal peripheral capillary oxygen saturation (SpO2) and arterial oxygen saturation (SaO2) measurements were recorded. The pharyngeal SpO2 was clinically evaluated. Also, variables associated with the SpO2 bias were evaluated for their association with the pharyngeal SpO2 bias.

**Results:**

The pharyngeal SpO2 bias was − 0.44% with − 1.65 to 0.78% limits of agreement. The precision was 0.62, and the accuracy was 0.05. The sensitivity to detect mild and severe hypoxemia was 100%, while specificity to minimize false alarm of hypoxemia was 100% for mild hypoxemia and 99.4% for severe hypoxemia. None of the studied variables were significantly associated with the pharyngeal SpO2 bias.

**Conclusion:**

The pharyngeal SpO2 has a clinically acceptable bias, which is less than 0.5% with high precision, which is less than 2%.

## Introduction

Monitoring oxygen saturation in shocked patients is a challenging nursing procedure as hypoperfusion greatly affects the accuracy of the peripheral capillary oxygen saturation (SpO2) measurements [1]. Consequently, the arterial oxygen saturation (SaO2) is used as an alternative to get more accurate measurements [2]. The frequent arterial blood sampling can cause iatrogenic anemia. So, selecting a pulse oximeter site that can be adequately perfused to improve SpO2 measurements will save the patient from the frequent blood sampling [3].

Finding a site for pulse oximeter measurements that is feasible and adequately perfused to get accurate SpO2 measurements is an interest of ongoing research [4–6]. The pharynx is considered an adequately perfused site [7]. A small number of research studies have been conducted to evaluate the feasibility and accuracy of using the pharyngeal SpO2 [8–10]. These studies did not provide a trusted data because of the inadequate sample size.

The clinical use of the pharyngeal SpO2 requires fulfillment of SpO2 performance criteria. These criteria are bias, precision, accuracy, sensitivity, and specificity [11, 12]. Bias is the difference between SpO2 and the SaO2 measurements. The standard deviation of this bias is the precision. Most manufacture instructions of pulse oximeter claim that precision of SpO2 is 2–4% [13, 14]. However, the clinically acceptable precision should not exceed 2% [15].

Accuracy according to the Food and Drug Administration (FDA) is the root mean square of bias and precision. Accuracy should not exceed the desired limit, which is 3% [15]. Sensitivity is the timely detection of hypoxemia. Hypoxemia in ICU is defined as SpO2 less than 90% [16]. Oxygen saturation below 90% ranges from 80 to 89% and less than 80%. Oxygen saturation below 80% is dangerous due to impaired mental function and the risk of tissue hypoxia [17, 18]. Sensitivity is estimated by the percentage of times that SpO2 detected hypoxemia, while specificity to hypoxemia is the percentage of times that SpO2 minimized the false alarm of hypoxemia [15].

The clinical performance of the SpO2 to estimate the SaO2 in critically ill patients and especially in shocked patients has yielded mixed results. There are multiple factors that can affect its clinical performance [19]. Patients' age, dark skin color, bilirubin level, hemoglobin (Hb) level, and oxygen therapy are factors that increase the bias of the SpO2 [13, 20]. Hypothermia and vasopressor drugs greatly increase bias by increasing the underestimation of the SaO2 [13]. Increased lactate also increases the underestimation of the SaO2 [15]. Hypoxemia severity is another factor that increases the bias of the SpO2. So, it is important to ensure that the used pulse oximeter has a high accuracy, especially when SpO_2_ is lower than 90% [21].

To use the pharyngeal SpO2 in the clinical practice, it should be evaluated against the SpO2 performance criteria. Also, factors that can affect its bias in clinical practice should be evaluated to avoid misleading oxygen saturation measurements. So, the current study was conducted. It is important to emphasize that this study used an experimental setup of a wrap oximeter sensor to an oropharyngeal airway (OPA) in order to evaluate the pharyngeal SpO2. There was no commercially available device to be used in this study.

## Objectives of the study

To clinically evaluate the pharyngeal oxygen saturation measurements in critically ill patients with shock.

1. Evaluate bias, precision, and accuracy of the pharyngeal SpO2 measurements in critically ill patients with shock.
2. Evaluate sensitivity and specificity of the pharyngeal SpO2 to hypoxemia in critically ill patients with shock.
3. Identify factors associated with the pharyngeal SpO2 bias in critically ill patients with shock.

## Design and methods

A prospective cohort research design was used in this study.

### Setting, sample, and recruitment

#### Setting

This study was conducted in five general ICUs at Alexandria Main University Hospital in Egypt. The total number of beds in these ICUs were 55 beds.

#### Sampling

MedCalc program version 19.6.4 was used to estimate the required sample size using a bias value of 0.5 ± 2% [15, 22] with power of 80% and alpha level equal to 0.05. The calculated sample size was 168 patients. Inclusion criteria were all patients with age from 18 to 60 years and admitted to the ICU with the diagnosis of any shock type. Patients who developed shock during their ICU stay were not included in this study. We included our shocked patients from the admission filing system. So, patients who developed shock during their ICU stay were missed. The exclusion criteria were patients with carbon monoxide poisoning, methemoglobinemia, recent use of intravenous contrasts for radiological studies, recent oral surgery or trauma within 14 days.

#### Recruitment

Patients were recruited from March to December 2020. Patients who were admitted to the ICUs with the diagnosis of shock were evaluated against the inclusion and exclusion criteria. Patients who met the inclusion criteria were included. At the time of inserting pharyngeal pulse oximeter, the patients' level of consciousness was assessed against the Glasgow Coma Scale (GCS) score. Patients with GCS score of 9 or less because of sedation or coma completed the study. Conscious (GCS more than 13) and semiconscious (GCS from 9 to13) patients were excluded. They were excluded because the use of oropharyngeal airway (OPA) in a conscious or semi-conscious patient with an intact gag reflex is contraindicated [23, 24].

### Study procedures

#### Measurement of the pharyngeal SpO2

**Fig. 1:**
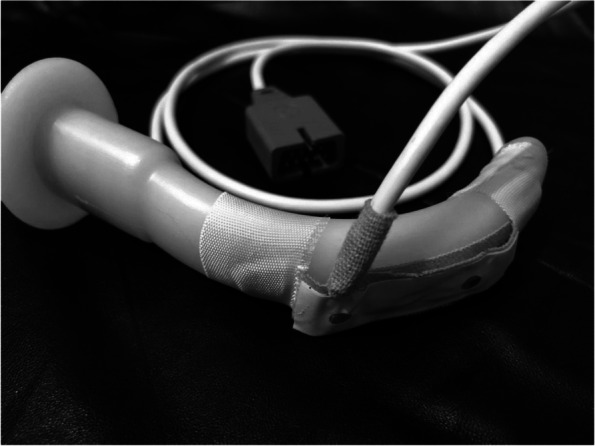
The pulse oximeter sensor attached to the posterior surface of the oropharyngeal airway

The selected pulse oximeter in this study was Reliable Nellcor Pulse Oximeter Probes. It has Conformite Europeenne (CE) and International Organization for Standardization (ISO) certificates. Its accuracy is ± 2% for oxygen saturation ranges from 90 to 100%, and ± 3% for oxygen saturation ranges from 70 to 89%. According to the FDA it is acceptable for pulse oximeter to be safe for use [25]. The appropriate size of Guedel OPA was selected. The appropriate size was measured from the incisors to the angle of the mandible. In our study, the pharyngeal pulse oximeter sensor was a pediatric wrap disposable oximeter sensor. The pulse oximeter sensor was attached to the posterior surface of the OPA using adhesive tape without covering the optical component. The optical component adhered to the pharyngeal surface after the OPA insertion [8] (Fig. 1).

Oropharyngeal suction was performed, then oral care with chlorhexidine (0.12% concentration) was done prior to OPA insertion. The patient's mouth was opened by crossed finger technique. Tongue blade was used to depress the tongue, then the OPA was inserted with the pharyngeal curvature till the flange comes to rest on the patient’s lips [23]. A clear waveform for the pharyngeal SpO2 was obtained after 30 s on the cardiac monitor, then oxygen saturation measurement was ready to be recorded.

#### Measurement of the SaO2

During four hours after the pharyngeal SpO2 insertion, the arterial blood gases (ABGs) sample was ordered by the physician at any time according to the patient's condition. At the same time of obtaining the ABG sample, the pharyngeal SpO2 measurement from the cardiac monitor was recorded manually. The SaO2 measurement was obtained from the result of the ABG analysis. If an arterial line was in use, the arterial blood sample was withdrawn from it into a heparinized syringe with 0.1 cc unfractionated heparin. Then, the arterial line was flushed, and the arterial blood pressure waveform was checked for accuracy on the cardiac monitor.

If the arterial line was not available, the arterial blood sample was obtained from the femoral artery because it is the best site for arterial puncture in shocked patients [26]. Air bubbles were immediately removed from the sample. The sample was immediately analyzed if the ABG analyzer was in the same ICU. If the ABG analyzer was not available, the sample was sent to the laboratory in an iced container.

#### Assessment of variables associated with the SpO2 bias

Variables associated with SpO2 bias were evaluated and documented by the researchers. Demographic variables, clinical variables, and therapeutic variables were used to categorize the variables. Age and gender were two demographic variables. Hypoxemia severity, shock type, body temperature, MAP, Hb level, lactate level, and bilirubin level were among the clinical variables studied. The type of oxygen therapy (venturi versus mechanical ventilator), the fraction inspiratory oxygen (FiO2), and the use of vasopressors were all considered therapeutic factors.We assessed if the patient received norepinephrine and/or epinephrine. The dose of norepinephrine and/or epinephrine ≥ 0.1 μg/ kg/min was considered a high dose which can affect SpO2 reading [27].

Only age, sex, and shock type were assessed from the chart at the time of enrollment in the study. The other variables were assessed immediately at the time of assessing oxygen saturation measurement. Blood pressure was measured from the arterial blood pressure device if present or the sphygmomanometer. Patients' temperature was measured from the rectal site. Lactate level and Hb level were obtained from the ABG sample analysis report. Bilirubin level was assessed from the laboratory results. The FiO2 was assessed from the venturi or the ventilator settings.

The researchers inserted the pharyngeal pulse oximeter sensor and compared the pharyngeal SpO2 with SaO2. Then the pharyngeal pulse oximeter sensor was removed. No negative outcomes from insertion of the pharyngeal pulse oximeter sensor were expected or actually occurred during the period of the study because it was attached to the OPA that was inserted according to its standard technique. Nurses and physicians in The ICU assessed the patients' oxygen saturation by the SaO2 measurement and their clinical decisions were based on its values not the pharyngeal SpO2.

### Data analysis

Data of this study were analyzed by SPSS version 25.0 (IBM Corp., Armonk, NY). Categorical variables were presented as number and percent. Continuous variables were presented as mean ± SD after testing its normality by the Shapiro–Wilk test. Bland Altman analysis and plot [28] were used to present bias of the pharyngeal SpO2 with the 95% limits of agreement.

The mean difference of pharyngeal SpO2 and SaO2 was considered to represent bias. The SD of the mean difference was considered to present precision. Root mean square of bias and precision was considered to represent accuracy. The clinically acceptable bias is less than 0.5%. The clinically acceptable precision is less than 2%. The clinically acceptable accuracy should not exceed 3% [2, 15]. Sensitivity was the percentage of times that pharyngeal SpO2 detect hypoxemia. Specificity was the percentage of time that the pharyngeal SpO2 does not alarm when there is no desaturation [15].

Multiple linear regression analysis was used to evaluate variables associated with pharyngeal SpO2 bias. We used backward regression analysis approach. We presented only the first step because none of the variables were found to be significantly associated with the pharyngeal SpO2 bias [29]. The selected variables were prior included in previous studies to affect SpO2 bias [15, 30, 31]. Statistical significance was set at *P* < 0.05.

### Ethical and research approvals

Research Ethics Committee, Faculty of Nursing, Alexandria University, Egypt approved this study before it was carried out. Approval to conduct the study was obtained from the hospital administrative authorities. Patients’ surrogates signed informed consent before participation in the study. The researchers ensured the privacy of the patients and confidentiality of the collected data. Data of the study were stored and managed by the first author on OneDrive storage space. It is accessed only by the four authors of this study. Data for this study can be shared with other researchers upon reasonable request from the first author and the Research Ethics Committee review and approval.

## Results

A total of 194 shocked patients were screened for eligibility. Twenty-six patients were excluded because of the following reasons. The researchers failed to obtain informed written consent from 11 patients. Nine patients had contraindications to OPA insertion. One patient had a carbon monoxide poisoning. The blood sample for ABG was not arterial in five patients. A total of 168 patients completed the study.

Table 1 presents patients' demographic and clinical characteristics. Patents' mean age was 39.59 ± 11.3 years, and 67.3% of patients were males. The highest frequent (66.1%) shock type was septic shock. The mean arterial pressure was 66.17 ± 5.05 mmHg and the mean lactate level was 1.12 ± 0.52 mmol/L. Norepinephrine was given to 45.8% of the patients, epinephrine was given to 7.6% of the patients, while epinephrine and norepinephrine were given to 3.1% of the patients. More than half (54.8%) of patients had an arterial line. The patients' mean SaO2 was 93.28 ± 7.33%, while the pharyngeal SpO2 was 92.85 ± 7.34%. In regard to the classification of oxygen saturation, 74.4% of patients has a normal saturation, 13.7% had mild hypoxemia and 11.9% had severe hypoxemia.

**Table 1:** Patients' demographic and clinical characteristics (*n* = 168)

Characteristics	Mean ± SD or n (%)
Age	39.59 ± 11.30 years
Sex	
Male	113 (67.3%)
Female	55 (32.7%)
Shock type	
Hypovolemic	28 (16.7%)
Cardiogenic	17 (10.1%)
Obstructive	6 (3.6%)
Neurogenic	3 (1.8%)
Septic	111 (66.1%)
Anaphylactic	3 (1.8%)
Temperature	37.80 ± 1.50 °C
MAP	66.17 ± 5.05 mmhg
Arterial line	
Yes	92 (54.8%)
No	76 (45.2%)
Lactate level	1.12 ± 0.52 mmol/L
Hb level	9.91 ± 1.81 g/dL
Bilirubin level	1.5 ± 0.9 mg/dL
Vasopressors	
Yes	95 (56.5%)
No	73 (43.5%)
Type of vasopressor	
Norepinephrine	77 (45.8%)
Epinephrine	13 (7.6%)
Norepinephrine and Epinephrine	5 (3.1%)
Oxygen therapy	
Venturi	19 (11.3%)
Mechanical ventilator	149 (88.7%)
FiO2	62.72 ± 22.61
SaO2	93.28 ± 7.33
Pharyngeal SpO2	92.85 ± 7.34
Oxygen saturation classification	
Normal	125 (74.4%)
Mild hypoxemia	23 (13.7%)
Severe hypoxemia	20 (11.9%)

*MAP* Mean arterial pressure, *Hb* Hemoglobin, *FiO2* Fraction inspiratory oxygen, *SaO2* Arterial oxygen saturation, *SpO2* Peripheral capillary oxygen saturation, Normal oxygen saturation: SaO2 from 90–100%, mild hypoxemia: SaO2 < 90- 80%, severe hypoxemia: SaO2 < 80%

**Fig. 2:**
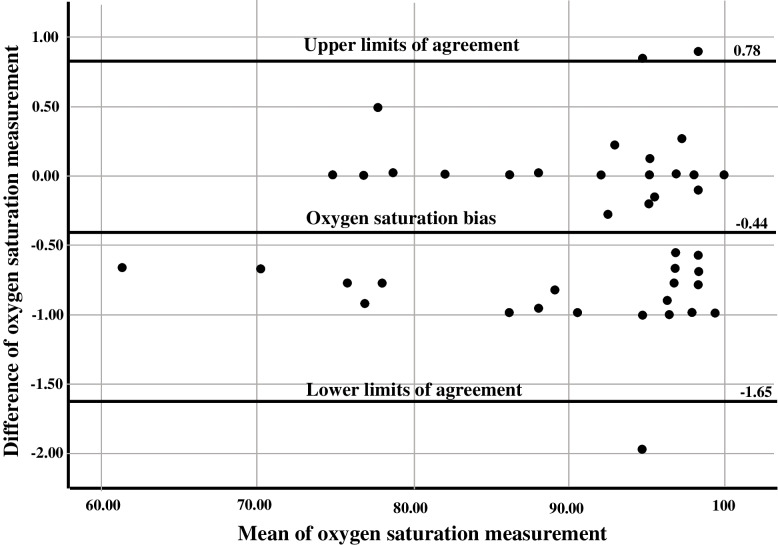
The Bland Altman plot for oxygen saturation measurements from arterial and pharyngeal sites

The Bland Altman plot for oxygen saturation measurements from the SaO2 and the pharyngeal SpO2 is shown in Fig. 2. The pharyngeal SpO2 bias was − 0.44%, and 95% limits of agreement ranged from − 1.65% to 0.78%. Table 2 illustrates measures of the pharyngeal SpO2 performance. Precision of the pharyngeal SpO2 was 0.62 and accuracy was 0.99. The sensitivity of pharyngeal SpO2 to detect mild and severe hypoxemia was 100%, while specificity to minimize false alarm of hypoxemia was 100% for mild hypoxemia and 99.4% for severe hypoxemia. Table 3 illustrates multivariable analysis to identify variables associated with the pharyngeal SpO2 bias. None of the demographic, clinical, or therapeutic variables were significantly associated with the pharyngeal SpO2 bias (all *p* < 0.05).

**Table 2:** Measures of the pharyngeal oxygen saturation performance

Performance of the pharyngeal SpO2	Values
Precision	0.62
Accuracy	0.99
Sensitivity to mild hypoxemia	100%
Sensitivity to severe hypoxemia	100%
Specificity to mild hypoxemia	100%
Specificity to severe hypoxemia	99.4%

*Precision* standard deviation of mean difference between SaO2 and pharyngeal SpO2 measurements, *accuracy* is root mean square of bias and precision, sensitivity: percentage of times that the pharyngeal SpO2 detect hypoxemia, *specificity* Specificity is the percentage of time the device does not alarm when there is no desaturation, *mild hypoxemia* SaO2 < 90- 80%, *severe hypoxemia* SaO2 < 80%

**Table 3:** Multivariable analysis identifying variables associated pharyngeal SpO2 bias

Variables	β	95% CI	*P*
**Demographic variables**			
Age	0.07	-0.13 to 0.21	0.63
Sex	-0.09	-0.51 to 0.26	0.50
**Clinical variables**			
Severity of hypoxemia	-0.01	-.15 to0.13	0.86
Shock type	-0.10	-0.14 to 0.08	0.53
Temperature	0.19	-0.02 to 0.23	0.12
MAP	-0.03	-0.08 to 0.01	0.08
Hb level	0.09	-0.07 to 0.13	0.54
Lactate level	-0.06	-0.40 to 0.28	0.69
Bilirubin level	0.02	-0.07 to 0.01	0.07
**Therapeutic variables**			
Oxygen therapy type	0.15	-0.29 to 0.89	0.31
FiO2	0.01	-0.01 to 0.02	0.41
Vasopressors use	-0.13	-0.20 to 0.52	0.39

*MAP* Mean arterial pressure, *Hb* Hemoglobin, *FiO2* Fraction inspiratory oxygen

## Discussion

The use of SpO2 measurements in shocked patients is a clinical challenge. The hypoperfusion state affects bias and precision of the oxygen saturation measurements. In this study, we selected the pharyngeal SpO2, which was expected to be adequately perfused than other conventional peripheral sites. Previous studies that evaluated the pharyngeal SpO2 measurements in hypo-perfused patients were old case study reports [8–10]. The current study was conducted with respect to the power analysis for the sample size to provide more generalized results.

The current study revealed that the pharyngeal SpO2 has a clinically acceptable bias with high precision. In contrast with another SpO2 site, a prior study revealed that finger SpO2 has an acceptable bias but low precision [22]. In this prior study, the use of vasopressor drugs in shocked patients was found to be a factor that was associated with high finger SpO2 bias [22]. In the current study, the pharyngeal SpO2 bias was not significantly associated with the use of vasopressor drugs.

Another prior study evaluated the accuracy and precision of SpO2 measurements in shocked patients with vasopressor drugs from finger and forehead sites [27]. The forehead SpO2 was less biased and more precise than finger SpO2, but in comparison with the current study values for accuracy and precision, the pharyngeal SpO2 is less biased and more precise than both the forehead and the finger sites.

The current study revealed that none of the demographic variables were significantly associated with the pharyngeal SpO2 bias. In previous study, gender was found to be a predictor for SpO2 bias [32]. The authors of this study suggested that this bias because of the small finger of the females or the lower Hb of the females than males. These factors were not found to be associated with pharyngeal SpO2 bias in the current study.

Regarding accuracy, the common manufacture literature claimed that the accuracy of SpO2 is ± 2–3% over the range of 70–100% oxygen saturation [33]. Also, they emphasized that the standard accuracy should be less than 3% to be clinically used [33, 34]. In the current study, the pharyngeal SpO2 displayed high accuracy that is less than 1% as compared to the golden standard which is the SaO2.

Even though the pharyngeal SpO2 has clinically acceptable bias, precision, and accuracy, it is worthy to comment that none of the studied patients experienced oxygen saturation less than 70%. So, the performance of the pharyngeal SpO2 measurements below 70% was not clinically evaluated in the current study. Pulse oximeters are usually calibrated to a range of saturation from 70 to 100% with an accuracy of 2% to 3%. This means that when pulse oximeter reading is lower than 70%, it may not be accurate when compared with the gold standard SaO2 measurements [2].

The current study evaluated the severity of hypoxemia as a factor associated with the pharyngeal SpO2 bias. The classification of hypoxemia in our study was mild hypoxemia, which ranged from less than 90% to 80%, and severe hypoxemia which ranged from less than 80% to 70%. We found that hypoxemia severity from 89 to 70% was not associated with the pharyngeal SpO2 bias. This result in contrast to a previous study result which evaluated the finger SpO2 measurements. It revealed lower accuracy with lower SaO2 with the same classification of hypoxemia according to the current study [12].

In addition, the current study revealed that the pharyngeal SpO2 was highly sensitive to detect hypoxemia and highly specific to exclude false hypoxemia alarm. This result is better than other SpO2 prior reported results. A prior study reported that SpO2 has a sensitivity of 92% to detect hypoxemia, and a specificity of 90% to exclude hypoxemia. In this prior study, hypoxemia threshold was 92% [35].

With respect to the current study sample, we can generalize that the pharyngeal SpO2 has clinically acceptable bias, precision, accuracy, sensitivity, and specificity in shocked patients. However, further studies are needed to clinically evaluate the pharyngeal SpO2 performance at SaO2 lower than 70%. Also, further studies are needed to evaluate factors that can affect pharyngeal SpO2 bias.

## Limitations

The pharyngeal SpO2 is not acceptable for individuals who cannot have an OPA, which is a major limitation of this study. None of the studied patients had oxygen saturation value < 70%. So, sensitivity and specificity of the pharyngeal SpO2 to severe hypoxemia are limited to the range from 70 to 79%. Hypoxemia was experienced by nearly a quarter of the patients. Therefore, the generalizability of pharyngeal SpO2 has clinically acceptable bias during hypoxemia is limited to 43 hypoxemic measurements. The study was not powered by repeated-measures tests. Repeated-measures were not utilized in this study because of the study approval restrictions. The hospital administrative authority concerned about patients' safety if the pharyngeal SpO2 was left in patients' pharynx for a longer time.

## Conclusion

Comparison between single measurement of the pharyngeal SpO2 and SaO2 in shocked patients revealed that the pharyngeal SpO2 measurement is accurate and precise. It has a clinically acceptable bias, high sensitivity, and high specificity to hypoxemia when hypoxemia ranges from 70 to 89%. Shocked patients should be assessed before the insertion of the pharyngeal pulse oximeter because it has multiple contraindications.

## Data Availability

The data and materials of the current study are not publicly available due to confidentiality reason but are available from the corresponding author on reasonable request.
